# *In vivo* dendritic cell targeting cellular vaccine induces CD4^+^ Tfh cell-dependent antibody against influenza virus

**DOI:** 10.1038/srep35173

**Published:** 2016-10-14

**Authors:** Satoru Yamasaki, Kanako Shimizu, Kohei Kometani, Maki Sakurai, Masami Kawamura, Shin-ichiro Fujii

**Affiliations:** 1 Laboratory for Immunotherapy, RIKEN Center for Integrative Medical Science, Yokohama, Kanagawa, Japan; 2 Laboratory for Lymphocyte Differentiation, RIKEN Center for Integrative Medical Science, Yokohama, Kanagawa, Japan

## Abstract

An induction of long-term cellular and humoral immunity is for the goal of vaccines, but the combination of antigens and adjuvant remain unclear. Here, we show, using a cellular vaccine carrying foreign protein antigen plus iNKT cell glycolipid antigen, designated as artificial adjuvant vector cells (aAVCs), that mature XCR1^−^ DCs *in situ* elicit not only ordinal antigen-specific CD4^**+**^T cells, but also CD4^+^ Tfh and germinal center, resulted in inducing long-term antibody production. As a mechanism for leading the long-term antibody production by aAVC, memory CD4^**+**^ Tfh cells but not iNKTfh cells played an important role in a Bcl6 dependent manner. To develop it for influenza infection, we established influenza hemagglutinin-carrying aAVC (aAVC-HA) and found that all the mice vaccinated with aAVC-HA were protected from life-threatening influenza infection. Thus, the *in vivo* DC targeting therapy by aAVC would be useful for protection against viral infection.

Successful vaccination against viral infection or cancer depends on the selection of the suitable form of antigen as well as the adjuvant. Adequate antibody responses of appropriate specificity elicited by vaccination are required to control and protect from many viral pathogens, such as influenza viruses, HIV and human papilloma virus (HPV)^1^. The most commonly used forms of vaccine antigens are inactivated virus, live attenuated virus, and recombinant viral proteins.

Depending on the type of adjuvant, some vaccines may enhance B cells directly, while others may enhance effective CD4^**+**^T cell responses. Development of synthetic anti-viral vaccines that trigger CD4^+^ T cell-dependent B cell immune responses has been attempted. However, even for targeting T cell-mediated antibody production, T cell responses are not optimally induced by commonly used adjuvants approved for human vaccine use, including alum- and oil-in-water emulsion-based adjuvants. Since vaccination with purified protein antigens plus conventional adjuvants typically results in the induction of only a modest antibody response by antigen-specific B cells with little or no T cell response, multiple immunizations may be required^1^. Therefore, the development of new vaccine adjuvants has been intensively explored to enhance the efficacy of weak antigens and broaden the immune response profile, leading to generation of high titer anti-viral antibodies. For such studies, the adjuvant has to be tested for its ability to increase overall antibody titer, as well as the amount of functional, e.g., neutralizing, antibodies and the quality of antibodies with high affinity for the antigen.

Invariant (i)NKT cells have a semi-invariant T cell receptor comprised of Vα14 in mice and Vα24 in human^2^,^3^. When activated by a glycolipid ligand, such as α-galactosylceramide (α-GalCer), they produce large amounts of IFN-γ and IL-4, suggesting that they can modulate immune responses. Indeed, several studies reported that iNKTfh cells could help B cells mount antigen-specific antibody responses^4^,^5^,^6^,^7^. Administration of a conjugate of lipid agonist and antigen protein initially activates iNKT cells and subsequently activates B cells that have captured the antigen, leading to substantially enhanced serological immunity to the cognate antigen^5^,^6^,^7^. On the other hand, we and others showed that co-administration of antigen-expressing cells plus α-GalCer or administration of antigen- and iNKT ligand-co-expressing syngeneic or allogeneic cells, so called artificial adjuvant vector cells (aAVC), generated antigen-specific CD8^+^ cytotoxic lymphocytes (CTL) through cross-presentation by dendritic cells (DCs) *in situ*^8^,^9^. In the current study, we examined whether the aAVC vaccine could also induce an efficient antibody response and, if so, whether it was CD4^**+**^Tfh cell- or iNKTfh cell-dependent. We also investigated the involvement of Bcl-6 in Tfh or iNKTfh cells that would provide help for B cells and assessed whether an aAVC vaccine could protect from influenza virus infection.

## Results

### Antigen-specific CD4^+^ T cell response induced by administration of allogeneic cells transfected antigen mRNA and loaded with α-GalCer depends on XCR1^−^ DCs

**Figure 1:**
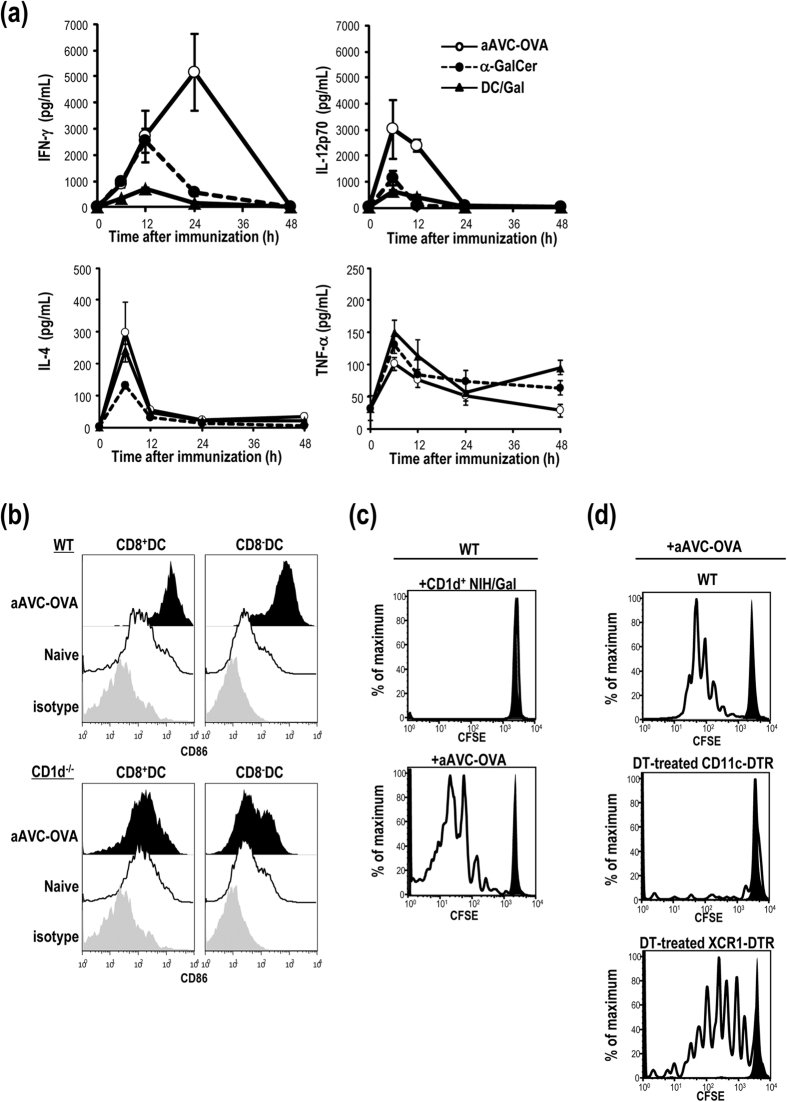
A vaccination with aAVC-OVA elicits antigen-specific CD4^+^ T cells via *in vivo* DC maturation. (**a**) Kinetics of serum cytokines after immunization of B6 mice at indicated time points with a single i.v. injection of α-GalCer (1 μg/mouse) or α-GalCer-loaded dendritic cells (DC/Gal) (1 × 10^6^ cells/mouse) or aAVC-OVA (5 × 10^5^ cells/mouse). Serum cytokines were measured by ELISA or Bio-Plex. (Mean±SEM, n = 4) (**b**) Expression of CD86 on CD8a^+^ and CD8a^−^ DC subsets in the spleen 16 h after an administration of aAVC-OVA in WT and CD1d^−/−^ mice. (n = 3) (**c,d**) *In vivo* MHC class II presentation and proliferation of OT-II after immunization with aAVC-OVA. CFSE-labeled OT-II cells were adoptively transferred into naïve WT (upper), DT-treated-CD11c-DTR (middle) or XCR1-DTR (lower) mice. One day later, mice were immunized with CD1d mRNA-transfected NIH3T3 cells loaded with α-GalCer (CD1d^+^NIH/Gal) (**c**) or aAVC-OVA (**c,d**) and proliferation of OT-II cells was assessed 3 days later. (solid, unimmunized control; clear: aAVC-OVA) Data are representative of 4 independent experiments.

We previously demonstrated the efficacy of a cellular vaccine comprised of OVA mRNA-transfected CD1d-allogeneic cells loaded with α-GalCer, which we termed OVA-expressing artificial adjuvant vector cells (aAVC-OVA)^8^. The aAVC-OVA expressed OVA protein (100–200 ng/5 × 10^5^ cells) as well as activated iNKT cells (Fig. S1). Following vaccination with an optimized aAVC-OVA, we first measured serum cytokines and detected higher amounts of IFN-γ and IL-12 than in mice immunized with free α-GalCer (free α-GalCer) or α-GalCer-loaded DCs (DC/Gal). However, the amounts of IL-4 and TNF-α were almost identical in all groups (Fig. 1a). These results suggest that the response is dominated by Th1 type cytokines with a small contribution by Th2 type cytokines. With regard to DC maturation, both CD8a^+^ and CD8a^−^ subsets of DCs in the spleen highly expressed CD86 16 h after aAVC immunization but not in CD1d^−/−^ mice (Fig. 1b). We previously reported that antigen-specific CD8^+^ CTL were generated after administration of aAVC-OVA though cross-presentation by CD8a^+^ CD11c^+^ DCs *in situ*^9^. In the current study, we found that antigen-specific CD4^+^ T cells are generated by endogenous DC in aAVC-OVA-immunized mice. To demonstrate this, C57BL/6 mice that had previously been injected with CFSE-labeled OT-II cells were immunized with aAVC-OVA or CD1d^+^allogeneic NIH3T3 loaded with α-GalCer (CD1d^+^NIH/Gal). OT-II proliferation was observed in mice given aAVC-OVA, but not in mice given CD1d^+^NIH/Gal (Fig. 1c). These results suggested that CD4^+^ T cells were not directly stimulated by NKT cells. XCR1 is exclusively expressed by CD8a^+^ CD11c^+^ MHCII^+^ DC in the spleen and this of DCs have been identified as responsible for cross-presentation of exogenous antigen^10^. When CD11c-DTR or XCR1-DTR-venus mice were treated with diphtheria toxin (DT), total CD11c^+^ DCs or CD8a^+^ subsets of DCs were depleted *in situ*, respectively. To assess which DC subsets can affect the activation of CD4^+^ T cells by the aAVC-OVA, CFSE-labeled OT-II cells were transferred into C57BL/6, and DT-treated CD11c-DTR or XCR1-DTR-venus mice. On the following day, we administered aAVC-OVA to these mice and, three days after a vaccination, we detected significant proliferation of OT-II cells in the vaccinated, WT mice and DT-treated XCR1 mice, but not in the DT-treated CD11c-DTR mice (Fig. 1d). These findings suggested that the XCR1^−^subset of DCs plays a central role in presenting protein antigen to CD4^+^ T cells.

### Efficient production of antibody by vaccination with aAVC-OVA rather than co-administration of antigen plus adjuvants

**Figure 2:**
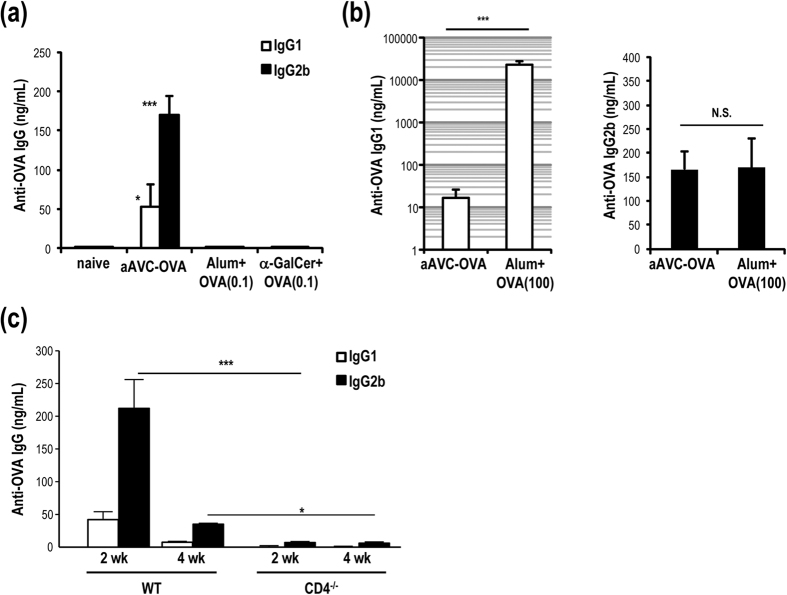
Th1-type antibody production by aAVC-OVA vaccination in a CD4^+^ T cell-dependent manner. (**a,b**) Serum anti-OVA antibody isotype at 2 weeks after immunization with alum plus OVA protein (0.1 or 100 μg) i.p., 500 ng α-GalCer plus OVA (0.1 μg) i.p. or aAVC-OVA (0.1 μg OVA protein-containing NIH3T3) i.v. (Mean±SEM, n = 4–5) *P < 0.05, ***P < 0.001. (aAVC-OVA vs others) (**c**) OVA-specific serum IgG1 and IgG2b antibody response were measured in WT mice and CD4^−/−^ mice 2 weeks after immunization with aAVC-OVA. (Mean±SEM, n = 9–12) *P < 0.05, ***P < 0.001.

To evaluate the antibody producing activity of several vaccine approaches, we used the same amount of OVA antigen (0.1 μg/mouse) for a direct comparison. C57BL/6 mice were immunized by co-injection of alum plus OVA protein, α-GalCer plus OVA or aAVC-OVA. Two weeks later, OVA-specific IgG1 and IgG2b serum antibody levels were much higher in the aAVC-OVA mice than in the other groups (Fig. 2a). We also performed dose response experiments in which we administered graded doses (1–100 μg) of OVA together with alum and found that the serum IgG response was optimal at the 100 μg dose (Fig. S2). When mice were immunized with 100 μg of OVA antigen plus alum, the IgG1 antibody levels were significantly higher in the alum plus OVA immunized mice compared to the aAVC-OVA immunized mice, however the levels of IgG2b were similar (Fig. 2b). Therefore, based on antigen dose (0.1 versus 100 μg), these results suggest that aAVC is 1000 times more potent than alum in inducing a Th1-type Ab response. To confirm that antibody production in aAVC-immunized mice depended on CD4^+^ T cells, we immunized WT or CD4^−/−^ mice and detected OVA-specific IgG1 and IgG2b antibody only in WT mice (Fig. 2c). These results indicated that the CD4^+^ T cell-mediated Ab responses in aAVC-OVA immunized mice are mostly of the Th1 type, but also include a smaller Th2 type Ab response.

### Antigen-specific multifunctional CD4^+^ T cells are generated in aAVC-OVA immunized mice

**Figure 3:**
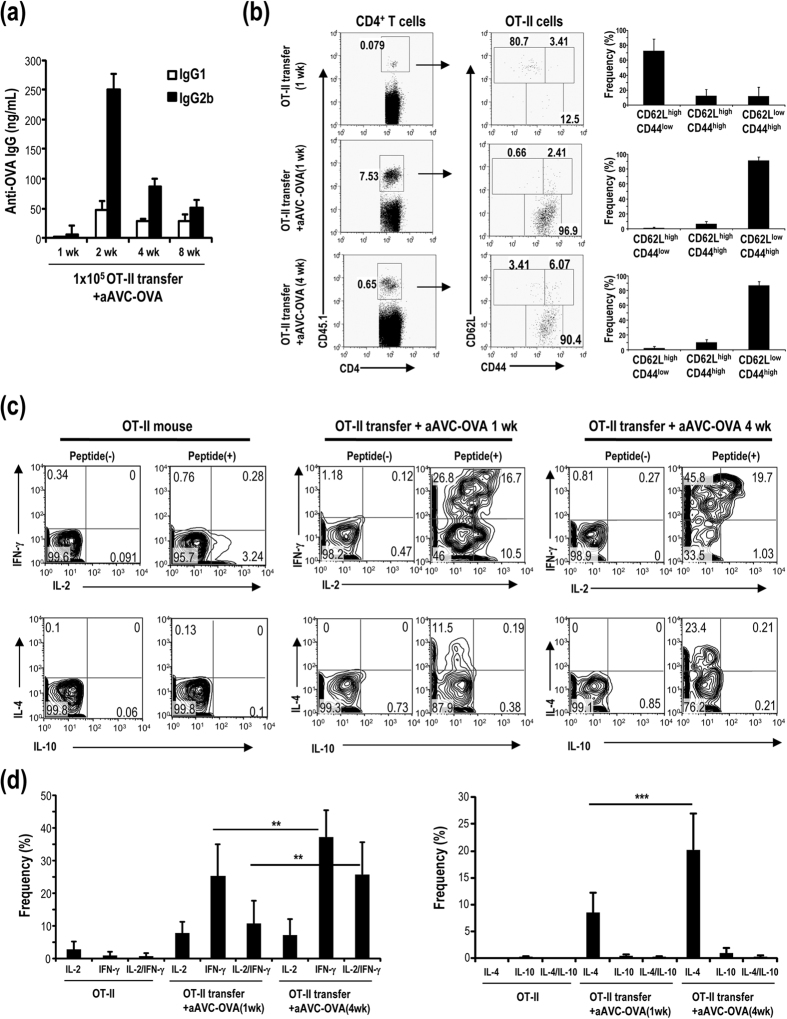
Antigen-specific CD4^+^ memory T cells induced by aAVC-OVA show the multi-functionality. (**a**) Kinetics of serum anti-OVA antibody isotype of OT-II transferred, aAVC-OVA immunized mice at indicated time points. C57BL/6 mice were transferred with 1 × 10^5^ OT-II cells and then immunized with 5 × 10^5^ aAVC-OVA 24 h later. OVA-specific IgG1 and IgG2b antibody levels were measured by ELISA 2, 4 and 8 weeks later. (Mean±SEM, n = 4–16) (**b–d**) Antigen-specific CD4^+^ T cells response after immunization of aAVC-OVA immunized mice. (**b**) As shown in (**a**) but the mice were immunized with or without 5 × 10^5^ aAVC-OVA. One week or 4 weeks after immunization, OT-II cells in spleen were analyzed for the frequency (left) and the expression of CD44 and CD62L (middle). The subsets were shown (right). (Mean±SEM, n = 4–9). (**c,d**) As shown in (**b**) but cytokine production by OT-II was analyzed by intracellular cytokine staining using IFN-γ-APC and IL-2-PE or IL-4-APC or IL-10-PE after stimulation with OVA peptide *in vitro* for 6 h gating on CD45.1^+^CD4^+^ cells. (Mean ± SEM, n = 4–9) **P < 0.01, ***P < 0.001.

To trace the CD4^+^ T cell response, C57BL/6 mice were transferred with a small number of OT-II cells (1 × 10^5^/mouse) and then immunized with aAVC-OVA one day later. The production of OVA-specific IgG1 and IgG2b antibody by these mice was comparable to that in non-OT-II transferred mice (Fig. 3a, compare to Fig. 2a), as was the preponderance of OVA-specific IgG2b over IgG1. These results indicated that the small number of transferred OT-II cells did not change the physiological response and thus could be used as a method to trace the T cell response. One week (effector phase) or 4 weeks (memory phase) after immunization with aAVC-OVA, OT-II cells in spleen were analyzed and the frequency of CD44^hi^CD62L^lo^ CD4^+^ effector memory T (T_EM_) was higher than the CD44^hi^CD62L^hi^CD4^+^ central memory T (T_CM_) cells (Fig. 3b). When we analyzed cytokine production by naïve OT-II cells, effector T cells (1 week) and memory T cells (4 weeks), the frequency of effector CD4^+^ T cells producing IFN-γ or both IFN-γ and IL-2 increased 1 week after an immunization and increased even further at 4 weeks (Fig. 3c upper and 3d left). Simultaneously, antigen-specific CD4^**+**^T cells producing IL-4, but not IL-10 were detected as a minor population (Fig. 3c lower and 3d right). Thus, immunization with aAVC-OVA primarily induces Th1 cells, although it can also induce IL-4-secreting Th2 cells. Even though we observed more IgG2b, IgG1 was still detectable, suggesting the induction of both types of Th-dependent antibodies. As for the other control groups, antigen-specific CD4^**+**^ T cells from mice injected with alum or unbound α-GalCer plus OVA were evaluated. These vaccines neither increased the number of antigen-specific CD4^**+**^ T cells, nor enhanced their cytokine production (Fig. S3) compared to aAVC-OVA, suggesting that the observed effects depend on the type and formulation of the adjuvants. These data indicated that CD4^**+**^T cells in mice given aAVC are multifunctional memory T cells, primarily Th1 cells but also including a low percentage of Th2 type CD4^**+**^T cells.

### Germinal centers are established by immunization with aAVC

**Figure 4:**
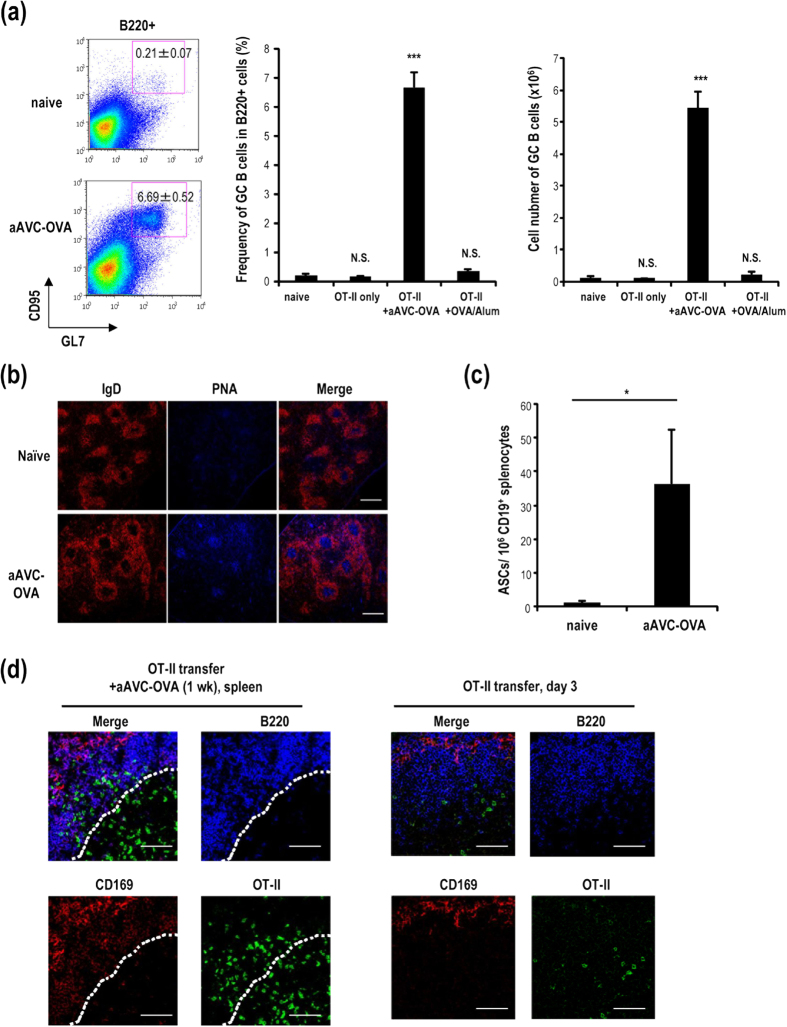
Germinal center formation following immunization with aAVC-OVA. (**a**) OT-II transferred mice were immunized with aAVC-OVA or Alum plus OVA protein (100 μg). Fourteen days later, germinal center (GC) formation in spleen was analyzed by flow cytometric analysis using B220-APC, CD95-PE and GL7-FITC. (Mean ± SEM, n = 4–6.) ***P < 0.001. (**b**) GC formation in spleen sections was verified by confocal microscopy using IgD-PE and PNA-Alexa 647. Scale bar, 200 μm (**c**) ELISPOT analysis of OVA-specific IgG-secreting splenic B cells. (Mean ± SEM, n = 4). *P < 0.05. (**d**) Localization of OT-II cells in OT-II transferred mice immunized with aAVC-OVA (left panel) or non-immunized (right panel) was analyzed using CD45.1-FITC, B220-APC, and CD169-PE 7 days after immunization or 3 days after a transfer of OT-II cells. The dotted line shows approximate location of the boundary between the B cell follicle and the T cell zone. All data are representative of at least four experiments. Scale bar, 50 μm.

It is well known that germinal center (GC) formation is required for affinity maturation and long-term antibody production^11^, therefore we analyzed the GC response in aAVC immunized mice. Fourteen days after immunization of WT mice with aAVC-OVA, germinal center B220^+^CD95^hi^GL7^hi^ B cells could be readily detected in spleen (Fig. 4a left). To monitor the CD4^**+**^T cells in the GC area, we again transferred OVA antigen-specific transgenic OT-II T cells one day before immunization with aAVC-OVA. The frequency and number of GC cells was increased in mice receiving OT-II cells and immunized with aAVC-OVA, but not in recipients of OT-II alone or OT-II plus OVA and Alum (Fig. 4a middle and right). The GCs formed in OT-II transferred, aAVC-OVA immunized mice were further examined by immunohistochemistry and confocal microscopy and shown to have the expected structure and GC markers, i.e., IgD^−^ and PNA^+^ (Fig. 4b). Next, we assessed the function of B cells using ELISPOT to quantify OVA-specific antibody production by CD19^+^ B cells isolated 14 days after immunization. OVA-specific antibody producing cells were detected in aAVC-OVA immunized mice but not in naïve mice (Fig. 4c). We also assessed the location of OT-II cells in OT-II-transferred mice and OT-II plus aAVC-OVA-immunized mice at day 3 or day 7 respectively. In mice receiving only T cells, there were very few OT-II cells in the B cell zone (Fig. 4d, right), whereas these cells were clearly detected in both PALS and the B cell zone in aAVC-OVA-immunized mice (Fig. 4d, left). These results indicate that some of the antigen-specific CD4^+^ T cells traffic to the B cell zone in aAVC-OVA-immunized mice.

### Antibody production in aAVC-OVA-immunized mice depends on CD4^+^ Tfh cells but not iNKTfh cells

**Figure 5:**
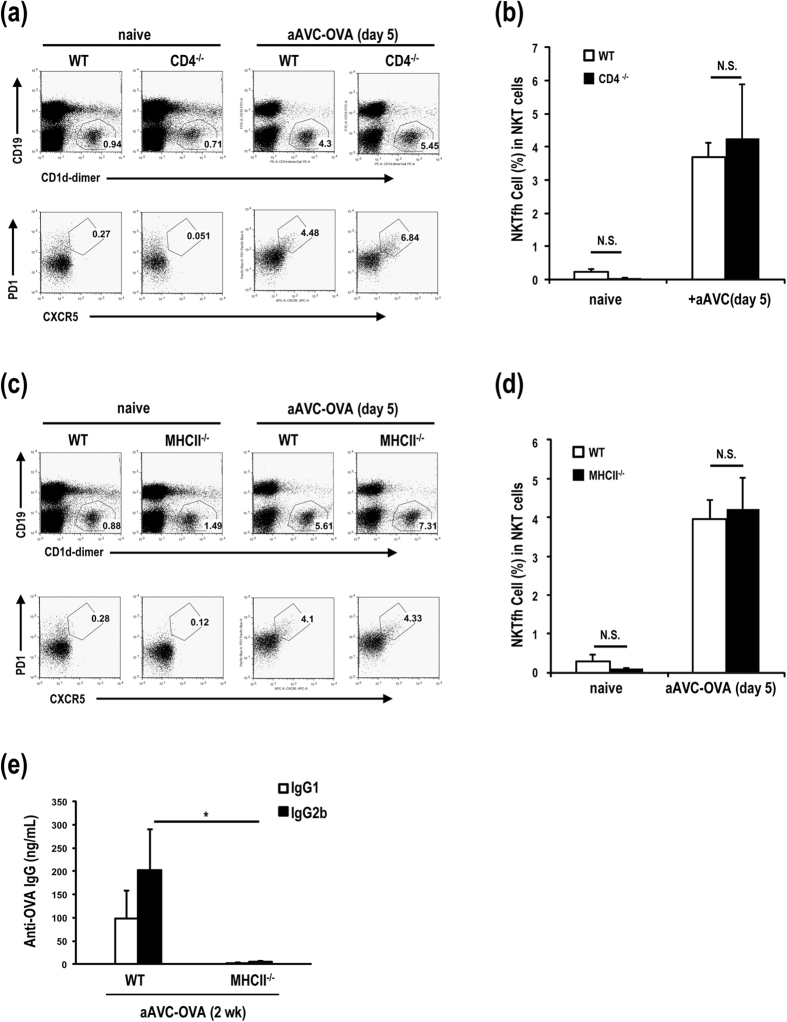
Antibody production in aAVC-OVA-immunized mice depends on CD4^+^ Tfh cells, but not iNKTfh cells. (**a,b**) The generation of iNKTfh cells in WT or CD4^−/−^ aAVC-OVA-immunized and naïve mice. WT mice and CD4^−/−^ mice were immunized with aAVC-OVA. Five days later, the frequency of iNKTfh cells in spleen were analyzed using CD19-FITC, CD1d-dimer-PE, PD1-VB, and CXCR5-APC. (Mean ± SEM, n = 5–6) (**c,d**) The frequency of iNKTfh cells in spleen were analyzed in WT or MHC II^−/−^ mice 5 days after immunization with aAVC-OVA. (**e**) OVA-specific serum IgG1 and IgG2b antibody response were measured in WT mice and MHC II^−/−^ mice 2 weeks after immunization with aAVC-OVA. (Mean ± SEM, n = 5–6) *P < 0.05.

A mechanism of T cell-dependent producing antibody response may apparently depend on the type of antigens. A recent study has reported that iNKTfh cells rather than CD4^**+**^Tfh cells are important for lipid antigen-specific antibody production^6^, therefore, we carefully investigated whether iNKTfh or CD4^+^ Tfh cells are important players mediating protein antigen-specific antibody in aAVC-immunized mice. As shown in Fig. 2c, OVA-specific IgG antibody production was not seen in CD4^−/−^ mice, although the number and frequency of iNKTfh cells in spleen is comparable in WT and CD4^−/−^ mice (Fig. 5a,b). MHC II^−/−^ mice lack CD4^+^ T cells but have the same number of iNKT cells as in WT mice (Fig. 5c), thus these mice provided another means to analyze the putative role of iNKTfh cells in the antibody response. As shown in Fig. 5e, both IgG1 and IgG2b were extremely low in aAVC-OVA-immunized MHC II^−/−^ mice, even though the number of iNKT cells was increased and, in particular iNKTfh cells were clearly generated (Fig. 5c,d). These data using CD4^−/−^ and MHC II^−/−^ mice indicate that iNKTfh cells have no significant role in antibody production after aAVC immunization.

### BCL6 in CD4^+^ Tfh cells plays a key role in antibody production

**Figure 6:**
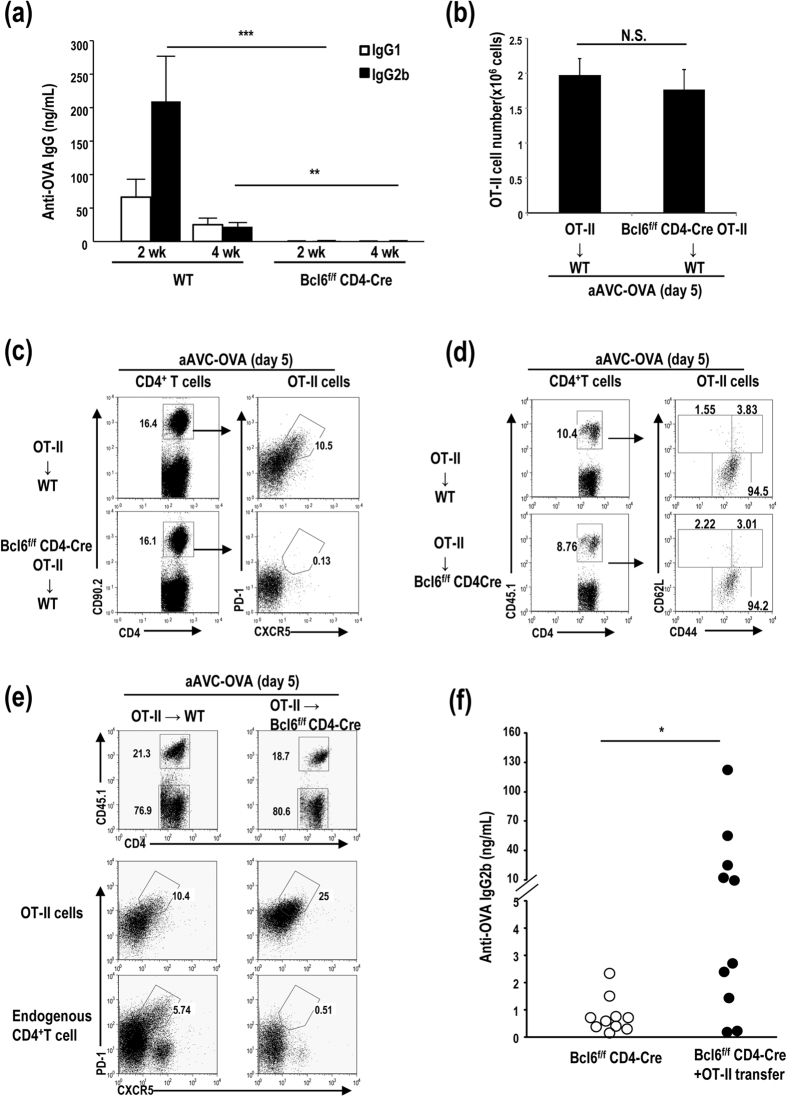
The antibody production of aAVC systems requires Bcl6 in Tfh cells. (**a**) Serum OVA-specific antibody isotype of WT and Bcl6^f/f^-CD4-Cre mice immunized with aAVC-OVA. OVA specific IgG1 and IgG2b levels in serum were examined 2 and 4 weeks later. (Mean ± SEM, n = 5–7) **P < 0.01, ***P < 0.001. (**b,c**) 1 × 10^5^ OT-II cells (CD90.2^+^) or Bcl6^f/f^-CD4-Cre OT-II cells (CD90.2^+^) were transferred into B6.PL mice (CD90.1^+^). On the following day, the mice were immunized with aAVC-OVA. The total cell number of OT-II (**b**) and the frequency of Tfh type OT-II cells (**c**) in spleen of vaccinated mice were assessed 5 days after immunization using CD4-FITC, CD90.2-PE, CXCR5-APC, and PD-VB. Data are representative of four mice. (**d,e**) 1 × 10^5^ CD45.1^+^ OT-II cells were transferred into WT or Bcl6^f/f^-CD4-Cre mice and immunized with aAVC-OVA on the following day. OT-II cells in spleen were analyzed for the expression of CD44 and CD62 (**d**) and Tfh phenotype (**e**) 5 days after immunization. Data are representative of four mice. (**f**) 1 × 10^6^ OT-II cells were transferred into WT or Bcl6^f/f^-CD4-Cre mice. On the following day, the mice were immunized with aAVC-OVA, and OVA-specific serum IgG2b levels were examined 2 weeks later. (n = 10), *P < 0.05.

We next focused on CD4^**+**^ Tfh. We assessed the CD4^**+**^ Tfh cells in C57BL/6 mice after aAVC-immunization and found that CD4^+^ Tfh cells increased after an injection of aAVC-OVA (Fig. S4). Then we asked whether the production of T-dependent antigen-specific antibody depends on the transcription factor Bcl6 at the level of B cells^12^,^13^ and/or CD4^**+**^T cells^14^,^15^,^16^. To evaluate the importance of Bcl6 in the CD4^+^ Tfh cells in our model, we assessed antibody production and the CD4^+^ T cell response in Bcl6^flox/flox^ CD4-Cre (Bcl6^f/f^CD4-Cre) and WT mice immunized with aAVC-OVA. OVA-specific IgG1 and IgG2b levels were elevated at 2 and 4 weeks after immunization in WT mice, but not Bcl6^f/f^CD4-Cre mice (Fig. 6a). Thus, Bcl6 expression in CD4^+^ T cells play an important role in a production of IgG1 and IgG2b antibodies. Next, to identify the requirement for antigen-specific Tfh cells in our model, we adoptively transferred CD90.2^+^OT-II or CD90.2^+^Bcl6^fl/fl^CD4-Cre OT-II (CD90.2^+^Bcl6^f/f^ OT-II) cells to WT mice, and immunized the recipient mice with aAVC-OVA. The number of CD90.2^+^OT-II and CD90.2^+^Bcl6^f/f^OT-II cells was equivalent (Fig. 6b). Then, we assessed whether a population of Tfh cells could be generated in a Bcl6-dependent manner. To do this, we assessed whether CD90.2^+^ CD4^+^CXCR5^hi^PD1^hi^Tfh could be generated in mice that had been administered with either CD90.2^+^OT-II cells or CD90.2^+^Bcl6^f/f^OT-II cells and then immunized with aAVC-OVA. CD90.2^+^CD4^+^CXCR5^hi^PD1^hi^Tfh cells were generated in the mice receiving CD90.2^+^OT-II cells but not CD90.2^+^Bcl6^f/f^OT-II cells (Fig. 6c). aAVC without antigen or aAVC without α-GalCer cannot expand the CD4^+^ OT-II Tfh cells (Fig. S5). Therefore, two components, foreign protein and NKT ligand on aAVC, which are taken up by host DCs, are required for antigen-specific CD4^+^ Tfh induction and antibody production. Next, OT-II cells were transferred to WT mice or Bcl6^fl/fl^-CD4-Cre mice. The phenotypic analysis of the transferred OT-II cells showed similar expression of CD4^+^CD62L^low^CD44^high^ T cells as an effector T cell phenotypic in these two groups 5 days after an immunization with aAVC-OVA (Fig. 6d). CD4^+^ Tfh cells at early activated phase expressed effector T cell markers. Antibody by aAVC were not detected in Bcl6^f/f^CD4-Cre mice, because endogenous CD4^+^ T cells were not converted into CD4^+^ Tfh cells (Figs 6a,e lower right), thus only the transferred OT-II cells would have the potential to help the antibody response. Then, OT-II cells were transferred to Bcl6^fl/fl^CD4-Cre mice to test if OT-II can rescue the antibody production. We detected the generation of CD45.1^+^CD4^+^CXCR5^hi^PD1^hi^Tfh from OT-II T cells in both WT and Bcl6^fl/fl^CD4-Cre mice (Fig. 6e, middle). Apparently, we detected OVA-specific IgG2b in OT-II cell-transferred, aAVC-immunized Bcl6^f/f^CD4-Cre mice, but not OT-II non-transferred Bcl6^f/f^CD4-Cre mice (Fig. 6f). These results suggested that Bcl6 in Tfh cells is required for the antibody production in aAVC vaccine.

### Secondary response elicited by boosting in an antigen-dependent manner

**Figure 7:**
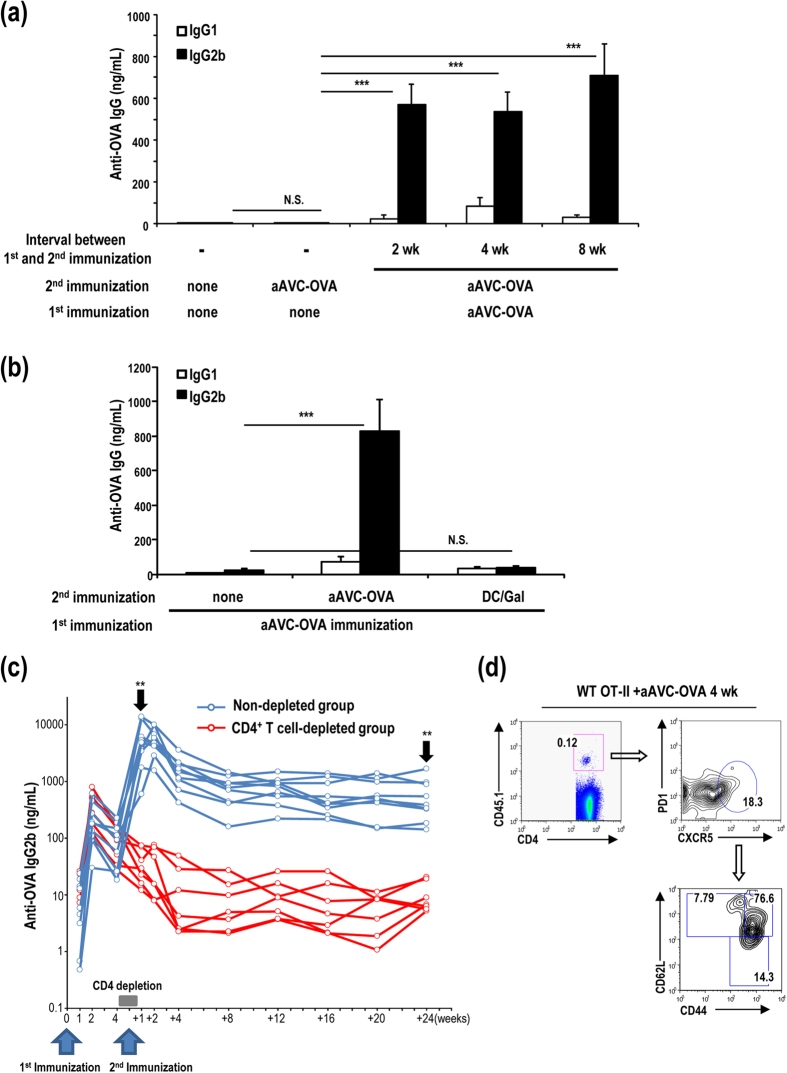
Long-term antibody production mediated by CD4^+^ memory T cells in mice vaccinated with aAVC-OVA. (**a**) WT mice were immunized with 5 × 10^5^ aAVC-OVA and boosted with aAVC-OVA 2 weeks, 4 weeks or 8 weeks later. Serum OVA-specific antibody isotype was measured in these mice 5 days after re-challenge. (Mean ± SEM, n = 5–7) ***P < 0.001. (**b**) As shown in (**a**) but these mice were rechallenged with 5 × 10^5^ aAVC-OVA or 5 × 10^5^ DC/Gal 2 weeks later. Fourteen days after boosting, serum OVA-specific antibody isotype was measured. ***P < 0.001. (**c**) As in (**a**) these mice were boosted with aAVC-OVA 35 days after the first immunization, with or without CD4^+^ T cell depletion (d28–35). Kinetics of serum OVA-specific IgG2b was analyzed at indicated time points. (n = 8–9) **P < 0.01 (non-depleted group vs CD4^+^ T cell-depleted group at +1 week and +24 weeks) (**d**) OT-II transferred mice were immunized with aAVC-OVA. Four weeks after immunization, OT-II cells in spleen were analyzed for the subset. Representative plots show the percentage of CD44^hi^CD62L^+^ cells after gating on CXCR5^+^PD-1^lo^ of transferred OT-II (CD4^+^CD45.1^+^).

Since antigen-specific CD4^**+**^T cells were generated by immunization with aAVC-OVA, we next examined the boosting effect. Serum antibody production was very low on day 5 after an immunization with aAVC-OVA (Fig. 7a). However, we detected higher antibody responses after boosting with aAVC-OVA at 2 weeks, 4 weeks or 8 weeks and analyzing 5 days later (Fig. 7a). On the other hand, when we boosted with syngeneic α-GalCer-loaded bone marrow-derived DCs (DC/Gal) rather than aAVC, we could not detect any increase in OVA specific antibody, suggesting that antibody production even in a secondary response was not dependent on iNKT cells (Fig. 7b). Thus, we demonstrated boosting effects of GC-dependent antibody production by repeated injections of the homologous aAVC-OVA, but not by boosting with iNKT ligand alone. In addition, the serum antibody levels in aAVC-OVA - aAVC-OVA immunized mice were maintained for at least 6 months (Fig. 7c). To test whether the boosting effect depends on CD4^**+**^T cells, we depleted CD4^**+**^T cells before and after boosting with aAVC-OVA (Fig. 7c). We only detected robust Ab production after aAVC-OVA boosting in the intact, but not in CD4^**+**^ T cell-depleted, animals. Some of OT-II cells express memory Tfh markers at maintenance phase, i.e., CXCR5^+^PD-1^low^CD62L^+^ 4 weeks after an administration of aAVC-OVA (Fig. 7d)^17^,^18^,^19^. Therefore, we conclude that CD4^**+**^Tfh cells play an essential role in OVA-antigen specific memory production of antibody.

### Antibody-mediated protection against influenza virus after vaccination with aAVC-HA

**Figure 8:**
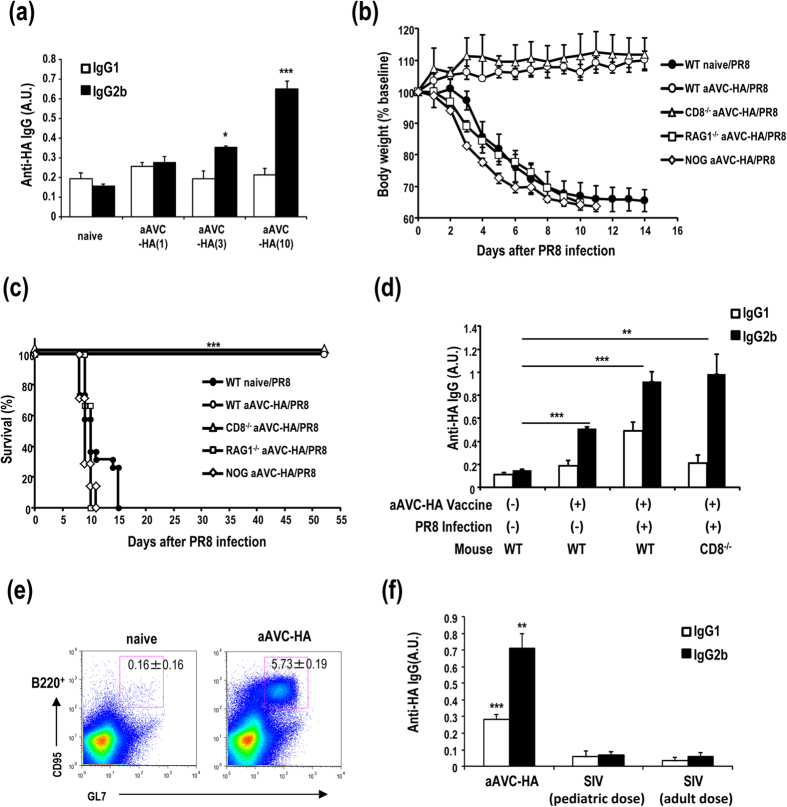
Vaccination with aAVC-HA protects against influenza virus infection. (**a**) HA-specific serum IgG1 and IgG2b antibody response were measured in WT mice 2 weeks after immunization with aAVC-HA (1, 3, or 10 μg HA mRNA-transfected aAVC-HA). (Mean ± SEM, n = 4) *P < 0.05, ***P < 0.001. (**b,c**) WT, CD8^−/−^, Rag1^−/−^ or NOG mice were vaccinated with 5 × 10^5^ aAVC-HA, and then challenged with lethal dose of PR8 influenza virus 2 weeks later. These mice were monitored for weight loss (**b**) and survival (**c**). WT mice without aAVC-HA vaccination and challenged with PR8 were examined as a control. (Mean ± SEM, n = 7–11) ***P < 0.001 (**d**) Serum anti-HA-specific antibody isotype was assessed by ELISA at day14 after aAVC-HA immunization or day 28 after aAVC-HA immunization, and subsequently PR8 infection. (Mean ± SEM, n = 4–7) **P < 0.01, ***P < 0.001 (**e**) GC formation was examined by flow cytometry analysis using B220-APC, CD95-PE, and GL-7-FITC. (Mean ± SEM, n = 4) (**f**) Comparison of the HA-specific antibody response to vaccination with aAVC-HA or the standard influenza vaccine (SIV). For these studies, the two standard doses of standard influenza vaccine (0.75 μg/kg for human pediatric dose and 0.3 μg/kg for human adult dose) or the aAVC-HA were administered to C57BL/6 mice. Serum HA-specific antibody was assessed by ELISA at day14 after vaccination. (Mean ± SEM, n = 4–5) **P < 0.01, ***P < 0.001.

We next investigated whether antibody induced by aAVC immunization can control influenza viral infection. We established aAVC containing the viral hemagglutinin (aAVC-HA) and then assessed antibody production by vaccination with 1, 3 or 10 μg HA mRNA-transfected aAVC to determine the optimal dose as 10 μg mRNA (Fig. 8a) (10 μg HA mRNA-transfected aAVC-HA expressed HA protein (625.91±76.2 ng/5 × 10^5^ cells) that was determined by western blot analysis). We assessed its ability to protect against influenza virus infection. C57BL/6 mice were immunized with aAVC-HA and then challenged with lethal dose of PR8 virus intranasally (i.n.) 2 weeks later. To evaluate the contribution of a CD8^+^ T cell-mediated anti-viral response to any observed protection, we used C57BL/6 and CD8^−/−^ mice. The antiviral response was evaluated by weight loss, survival and HA-antigen specific antibody production. Both WT and CD8^−/−^ mice vaccinated with aAVC-HA maintained their weight (Fig. 8b) and all the mice in these two groups survived long-term (Fig. 8c). However, unvaccinated mice and vaccinated Rag1^−/−^ or NOD/Shi-*scid*/IL-2Rγ^null^ (NOG) mice succumbed to the infection (Fig. 8c). Anti-HA antibodies were detected in aAVC-HA immunized mice and aAVC-HA immunized, PR8 virus-infected mice, and the absence of CD8^+^ T cells had no appreciable effect on this response (Fig. 8d). Similar to the aAVC-OVA immunized mice (Fig. 4a), GC formation was also detected in aAVC-HA-immunized mice (Fig. 8e). Next, we compared the aAVC-HA to the standard influenza vaccine (SIV). We used the two standard doses of the standard influenza vaccine (0.75 μg/kg for children and 0.3 μg/kg for adults) and aAVC-HA. As shown in Fig. 8f, immunization with aAVC-HA leads to a much better HA-specific antibody response than with the current standard influenza vaccine (Anti-HA IgG2b production: aAVC-HA, 0.713±0.088; SIV for pediatric dose, 0.0702±0.020; and SIV for adult dose, 0.0588±0.020 (A.U.)).

## Discussion

Weak and short duration of antibody production may be induced when B cells capture antigen and are stimulated in a GC independent manner^20^, thus probably being insufficient for the vaccine. In the current study, we have characterized the long-lasting cognate antigen-specific antibody production by aAVC vaccine in which antigen-specific CD4^**+**^Tfh cells elicited by iNKT cell-matured DCs were probably engaged in cognate interactions with B cells, resulting in formation of early GCs and strong antibody production. The formation of GCs in secondary lymphoid organs is known to be essential for Ig class switching, somatic mutation, affinity maturation, and long-lasting antibody responses^11^. We also demonstrated that iNKT cell-licensed DCs could drive the CD4^**+**^Tfh cells into the B cell region to generate GC and memory CD4^**+**^T cells in lymphoid tissues. Finally, we demonstrated the importance of CD4^**+**^Tfh cells rather than iNKTfh cells for inducing efficient antibody production after aAVC vaccination and could show protection using this vaccination strategy in an influenza infection model.

Among three types of mice deficient in GC formation, LTα^−/−^ mice and Lyn^−/−^ mice showed affinity maturation and long-lasting antibody production, but Bcl6^−/−^ mice did not^21^,^22^,^23^,^24^,^25^,^26^. Since Bcl6 plays a key role in efficient long-term antibody production, we analyzed the respective roles of Tfh and iNKTfh cells, as well as Bcl6 in Tfh and iNKTfh cells. It was reported that expression of CD1d on antigen-specific B cells is not required for the formation of iNKTfh cells^6^, suggesting that the formation of iNKTfh cells occurrs before provision of help to B cells. Immunization with protein Ags mixed with α-GalCer elicited detectable specific antibody in MHC II^−/−^ mice, suggesting the iNKT cell-mediated Ab production^4^. However, as shown in Fig. 7c, in our system long-term antibody production depends on CD4^**+**^T cells. Thus, memory CD4^+^ Tfh cells generated by *in vivo* DC maturation have the capacity to efficiently induce an antibody response. With regard to GC formation and function, GCs induced by NP-α-GalCer had limited affinity maturation, similar to T cell-independent GCs formed after polysaccharide (NP-Ficoll) immunization^27^,^28^. Thus, iNKTfh cells have functions similar to Tfh cells in terms of CXCR5^+^ GC formation and Bcl6 dependency, but their help is intrinsically limited compared with conventional Tfh cell help. This may be reflected in the fact that the B cell antigen response elicited by iNKTfh cells might be mostly an extrafollilular response and therefore of short duration and weaker than the response induced by Tfh cells. On the other hand, iNKT cells-licensed DC functions led to improved priming of Th cells and also enhanced provision of MHC class II-restricted cognate help for B cells^29^. Therefore, in terms of antibody production, an indirect response is more powerful than a direct response.

Influenza virus is a member of the orthomyxoviridae family that contain segmented negative-sense single-stranded RNA genomes^30^. Immunity against influenza virus is largely mediated by neutralizing antibodies that target the major surface glycoprotein hemagglutinin (HA)^31^, in particular the immunodominant head region of HA^32^. However, because of the high mutation rate in the HA head region, the virus undergoes continuous antigenic drift, and therefore the antiviral antibodies generated by immunization or natural infection are only effective against limited viral strains^33^,^34^,^35^,^36^. On the other hand, the stem region is less permissive for mutations than the head and antibodies that target conserved antigenic sites of the membrane-proximal HA stem region exhibit much broader neutralizing activity and can target strains within entire viral subtypes and across divergent influenza types^36^,^37^,^38^,^39^. Pappas *et al*. isolated 197 stem-specific antibodies from memory B cells or plasma cells following vaccination or infection. They found that 40% of the antibodies used the VH1-69 gene and arise from repeated stimulation in germinal centers^40^. As shown in Fig. 4a,e, antigen-specific antibody production apparently depended on the germinal center. As a future study, if we can select the optimal stem region antigen, we could make optimal antigen mRNA and develop a universal HA antigen-expressing aAVC. We could easily modify this protein and the manufacturing process for this vaccine has a shorter timeframe than others, which would be valuable during a pandemic response. The aAVC vaccine thus holds great promise as a potential broad spectrum prophylactic or therapeutic agent and for the development of a universal influenza or other viral vaccine.

## Methods

### Mice and cell lines

Pathogen-free 6-8-week-old C57BL6/J and Rag1^−/−^ mice were purchased from CLEA Japan. NOD/Shi-*scid*/IL-2Rγ^null^ (NOG) mice were purchased from the Central Institute for Experimental Animals (Kawasaki, Japan). CD4^−/−^ mice, CD8^−/−^ mice, MHC Class II^−/−^ mice and OT-II TCR-transgenic mice were purchased from Jackson Laboratory. CD1d^−/−^ mice were provided by Dr. Luc Van Kaer (Vanderbilt Univ., TN, USA). Bcl6^f/f^-CD4-Cre mice were provided by Dr. T. Takemori (RIKEN). Ly5.1 congenic OT-II mice were generated by cross/backcross breeding of OT-II with B6. Ly5.1 mice and screening for the presence of Vβ2 and Ly5.1 and absence of Ly5.2 expression by flow cytometry. Bcl6^f/f^-CD4-Cre OT-II mice were generated by cross breeding of OT-II with Bcl6^f/f^-CD4-Cre mice. CD11c-diphtheria toxin receptor (DTR) transgenic (CD11c-DTR/GFP) mice and XCR1-DTR-venus mice (XCR1-DTR) were generously provided by Dr. D.R. Littman (New York Univ., New York, NY) and Dr. T. Kaisho (Wakayama Univ., Wakayama, Japan). All mice were maintained under specific pathogen-free conditions and all animal experiments were performed in accordance with relevant guidelines and regulations. All of procedures and animal protocols was approved by the Animal Research Committee at RIKEN (permit number 28–020). NIH3T3 cell line was obtained from the RIKEN CELL BANK.

### Reagents

The following anti-mouse mAbs were purchased from BD Biosciences, eBioscience, R&D Systems, or BioLegend: anti-CD4 (GK1.5), -CD8a(53–6.7), -CD11c(HL3), -CD44(1M7), -CD62L(MEL-14),-CD45.1(A20), -CD86(GL-1), -CD90.2(30-H12), -CD95(15A7), -CD169(3D6.112), -CD279(29F.1A12), - IgD (17–26),- GL7 (GL7),-CXCR5(2G8),-IFN-γ(XMG1.2),-IL-2(JES6–5H4),-IL-4(11B11),-IL-10(JES5-16E3), CD1d-dimerix. Anti-OVA mAb (IgG1:2D11, IgG2b:1E7) was purchased from SantaCruz. Anti-mouse IgG1-HRP, anti-mouse IgG2b-HRP, and lectin PNA-Alexa647 were purchased from Invitrogen. Endotoxin-free chicken egg albumin was purchased from Seikagaku Corporation.

A FACSCalibur or FACSCanto II instrument and CellQuest, Diva (BD Biosciences), or FlowJO (TreeStar) software were used for analysis. For depleting CD4^**+**^T cells, anti-CD4 mAb (GK1.5) was prepared in our laboratory from s hybridoma. α-GalCer was synthesized and provided by Dr.Y. Ishii (RIKEN). Wild-type influenza A/PR/8/34 was purchased from ATCC. Recombinant H1N1 (A/Puerto Rico/8/1934) HA protein and anti-HA monoclonal antibody (2F1A7) was purchased from Sino Biological. H1N1 virus solution used for ELISA antigen was purchased from Denka Seiken, Japan. Standard influenza vaccine (SIV) was produced and purchased from Kaketsuken, Japan.

### Cell preparation

Mononuclear cells from the spleen, liver, lung, bone marrow, and lymph node were isolated as previously described. In brief, splenocytes were obtained by pressing the spleen through 70 μm cell strainer, and erythrocytes were lysed with ACK lysing buffer (Life Technologies), followed by two washes with RPMI1640. Lung and liver cells were floated on Percoll gradients (40/70%; Amersham Pharmacia Biotech) and centrifuged for 30 min at 900 × g to separate the mononuclear cells. In some experiments, CD19^+^ cells were purified with CD19 magnetic beads (Miltenyi Biotec).

### Preparation of aAVC-OVA and aAVC-HA

The OVA plasmid used for this study and generation of mRNA were described previously^9^. For the hemagglutinin (HA), the coding sequence from influenza A virus subtype H1N1 (GenBank Accession Number: CY045764) was generated by gene synthesis (Takara) and cloned into the *Hin*dIII and *Bam*HI sites of pGEM-4Z vector (Promega). The resultant plasmid was then linearized with *Bam*HI and purified with QIAquick PCR Purification Kit (Qiagen). mRNA for HA was synthesized by *in vitro* transcription with the above linearized template using mMessage mMachine T7 Ultra Kit (Ambion) and purified using RNeasy Mini/Midi Kits (Qiagen). The integrity of the mRNA was analyzed by agarose gel electrophoresis on denaturing conditions.

To load NIH3T3 cells with α-GalCer, the cells were cultured for 48 h in the presence of 500 ng/mL α-GalCer and then washed three times before electroporation. α-GalCer-loaded NIH3T3 cells (NIH3T3/Gal) were transfected with antigen mRNA together with murine CD1d mRNA using a square pulse electroporator (BTX ECM630, Harvard Apparatus) as described^41^. For transfection, electroporation was carried out using 5 μg of mCD1d mRNA and 10 μg of OVA or 10 μg of HA mRNA by a single 500 V/3 msec pulse. Immediately after electroporation, the cells were transferred to culture medium in the presence of 500 ng/ml of α-GalCer.

### Examination of aAVC-induced OT-II proliferation

To analyze OVA presentation, mice were adoptively transferred with 1 × 10^6^ CFSE-labeled CD45.1^+^OT-II cells and immunized on the following day with or without 5 × 10^5^ aAVC-OVA. OT-II cell proliferation in the spleen was monitored by dilution of CSFE 3 days later. In some experiments, CD11c-DTR/GFP mice or XCR1-DTR mice that had been treated with 4 ng/g/day or 20 ng/g/day diphtheria toxin (DT) (Sigma-Aldrich) respectively for 5 days from 2 days before aAVC-OVA immunization.

### Cytokine secretion assays and intracellular staining

Cytokine production by CD45.1^+^CD4^+^ OT-II T cells was determined using a protocol for intracellular staining. In brief, splenocytes were incubated in the presence of GolgiPlug (BD Bioscience) for 6 h, with or without 10 μM OVA_323–339_ peptide (ISQAVHAAHAEINEAGR), and then incubated with antibodies for surface markers. The cells were fixed and permeabilized with Cytofix/Cytoperm (BD Bioscience) and stained with anti-IFN-γ, anti-IL-2, anti-IL4, and anti-IL10 mAb.

### Immunofluorescence microscopy

Spleens were embedded in OCT compound (Sakura Finetek). Frozen sections (12 μm thick) were fixed with acetone and stained with the indicated antibodies. The following antibodies were used; anti-CD45.1, anti-B220, anti-CD169, and anti-IgD. The Abs were visualized with appropriate secondary antibodies conjugated with FITC, Cy3, or Cy5. For staining of germinal centers and nuclei, lectin PNA-Alexa 647 and DAPI were used, respectively. Immunofluorescence images were obtained using a confocal laser scanning microscope (TCS SP2; Leica) and images were transferred to Adobe Photoshop 7.0.

### ELISA

OVA-specific IgG1 and IgG2b levels were determined by solid phase immune assay. A high affinity-type 96 well flat plate was incubated with 100 μg/mL OVA in 50 mM carbonate buffer (pH 9.0) at 4 °C for 12 h. After washing three times with 0.05% Tween20 containing TBS buffer, plates were blocked for 1 h with Super Block blocking Buffer (Amersham Biotech) at room temperature (RT). After a single wash, 100 μL of serum from each mouse was incubated for 3 h at RT and then the wells were washed three times. Plates were then incubated with anti-mouse IgG1-HRP (1:1000) or anti-mouse IgG2b (1:2000) for 1 h at RT. After three washes, 100 μL of TMB substrate was added in each well, the reaction was stopped with 0.5 M H_2_SO_4_, and the absorbance at 450 nm was read immediately. Mouse IgG1 and IgG2b OVA antibodies were used as a standard for Ig concentration. For detection of anti-HA antibodies, H1N1 virus was used as an HA antigen. Virus solution was diluted in 50 mM Carbonate buffer (pH 9.0) and incubated in the plates at 4 °C for 12 h.

Serum cytokines were measured by ELISA (R&D) for IL-2 and IFN-γ, Bio-Plex (Bio-Rad) for IL-4, IL-12p70 and TNF-α.

### B cell ELISPOT assay

The number of OVA-specific antibody secreting cells was detected using B-cell ELISpot (MabTech), according to the manufacturer’s instructions. In brief, 96 well ELISpot assay plates were coated with 100 μL of 1 μg/mL OVA at 4^o^ C overnight. After five washes, plates were blocked with 10% FCS containing culture medium for 1 h at RT. CD19^+^ cells were purified from splenocytes of aAVC-OVA immunized mice (2weeks) and 1 × 10^6^ cells were seeded in each well and cultured for 24 h. After five washes, plates were incubated with biotinylated anti-IgG and followed by washing and incubation with streptavidin-HRP. The reactions were developed using TMB substrate and stopped by washing in water. Spots corresponding to OVA-specific antibody secreting cells were counted in each well. The results were represented as Ab-secreting cells per million CD19^+^ splenocytes (ASCs/10^6^ CD19^+^ splenocytes).

### Influenza infection

Mice were immunized with or without 5 × 10^5^ aAVC-HA, 2 weeks prior to virus infection. Mice were infected with 1 × 10^3^ pfu PR8 influenza virus in 50 μl of PBS intranasally and examined for body weight loss and survival. In some experiments, sera of infected mice were collected at day 14 after influenza virus infection.

### Statistical analysis

The survival curves of the treatment groups were plotted using the Kaplan-Meier estimates and compared through log-rank analysis. The Mann-Whitney U test was used for the statistical analysis of the remaining data. P < 0.05 was considered statistically significant.

## Additional Information

**How to cite this article**: Yamasaki, S. *et al*. *In vivo* dendritic cell targeting cellular vaccine induces CD4^+^ Tfh cell-dependent antibody against influenza virus. *Sci. Rep*. **6**, 35173; doi: 10.1038/srep35173 (2016).

## Supplementary Material

Supplementary Information
