# Supplementary material for: In vivo dendritic cell targeting cellular vaccine induces CD4+ Tfh cell-dependent antibody against influenza virus
Source: Sci Rep. 2016 Oct 14;6:35173. doi: 10.1038/srep35173 (PMC5064395; doi:10.1038/srep35173)
Supplement: Supplementary Information [file srep35173-s1.pdf]

Supplemental Information

In vivo dendritic cell targeting cellular vaccine induces $CD4^+$ Tfh cell-dependent antibody against influenza virus

Satoru Yamasaki, Kanako Shimizu, Kohei Kometani, Maki Sakurai, Masami Kawamura, and Shin-ichiro Fujii

Figure S1. Characterization of aAVC-OVA

(a) aAVC-OVA were established using NIH3T3 as vector cells by co-electroporation with OVA and CD1d mRNA and loading with α-GalCer. Eight hours after electroporation, CD1d expression **(**Red, aAVC-OVA; Black, isotype**)** **(b)** and the amount of OVA protein (ng/5x10^5 cells) **(c)** were assessed by flow cytometry and ELISA (ITEA Inc.), respectively. In addition, the NKT cell stimulating capacity of aAVC-OVA was examined by co-culturing with the Vα14 iNKT cell hybridoma 1.2 [kindly provided by Dr. M. Kronenberg (La Jolla Inst., La Jolla, CA)] or spleen cells and measuring IL-2 **(d)** or IFN-γ production **(e)**, respecttively.

Figure S2. Serum anti-OVA antibody titers induced by vaccination with alum plus OVA protein

Serum IgG1 (a) and IgG2b (b) anti-OVA antibody levels were assessed by ELISA at 2 weeks after immunization with alum plus graded doses of OVA protein (1, 10, or 100 μg) i.p. (Mean ± SEM, n=5)

Figure S3. Effector CD4 T cells induced by aAVC or other vaccine formulations

(a, b) C57BL/6 mice were transferred with 1 x 10^5 OT-II cells and then injected i.p. with

alum plus 100 μg OVA protein, 500 ng α-GalCer plus 100 μg OVA, or $5x10^5$ aAVC-OVA i.v. 24h later. One week after an immunization, spleen cells were isolated and the frequency (left) and absolute number (right) of OT-II cells in the vaccinated mice were analyzed using CD45.1-FITC and CD4-APC. (Mean ±SEM, n=4) *P<0.05, **P<0.01. (c) As shown in (a), but spleen cells from each group of mice were stimulated with OVA peptide *in vitro* for 6h and cytokine production by OT-II cells was analyzed by intracellular cytokine staining using IFN-γ-APC and IL-2-PE or IL-4-APC and IL-10-PE after gating on $CD45.1^+CD4^+$ cells.

Figure S4. The frequency of $CD4^+$ Tfh cells in aAVC-OVA-immunized mice

(a, b) WT mice were immunized with aAVC-OVA. Five days later, the frequency of Tfh cells among $CD4^+$ T cells in spleen was analyzed using CD4-PerCP/Cy5.5, PD1-VB, and CXCR5-APC. (Mean ±SEM, n=5-6) *P<0.05.

Figure S5. Two components of aAVC-OVA for an induction of $CD4^+$ Tfh

C57BL/6 mice were transferred with 1 x 10^5 OT-II cells and then immunized with 5 x 10^5 CD1d-NIH3T3/Gal (aAVC without OVA), CD1d-NIH3T3-OVA (aAVC without α-GalCer) and aAVC-OVA 24 h later. The total number of OT-II cells (a) and $OT\text{-}II^+$ Tfh

cells (b) were assessed 5 days after immunization. (Mean ± SEM, n=4) *P<0.05, ***P<0.001.

Figure S1

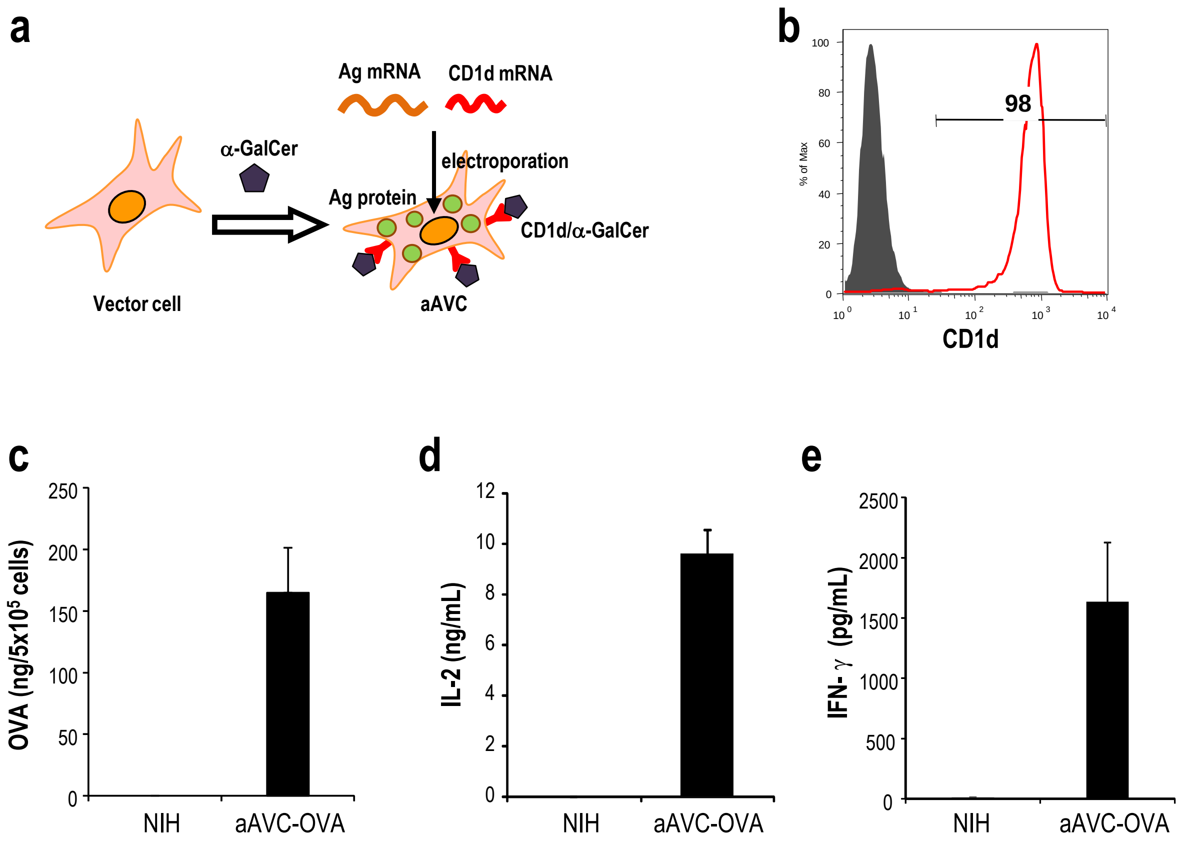

a
Ag mRNA
CD1d mRNA
α-GalCer
electroporation
Ag protein
CD1d/α-GalCer
Vector cell
aAVC
b
98
% of Max
CD1d
c
OVA (ng/5x10^5 cells)
NIH
aAVC-OVA
d
IL-2 (ng/mL)
NIH
aAVC-OVA
e
IFN-γ (pg/mL)
NIH
aAVC-OVA

Figure S2

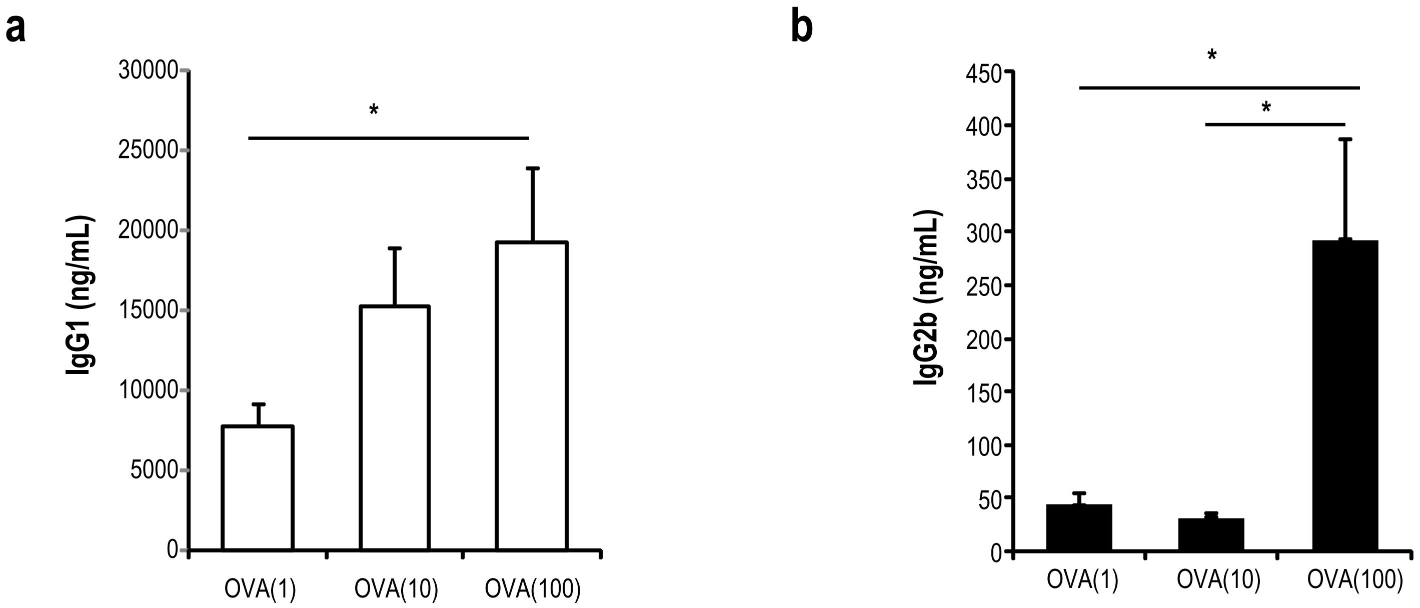
a
30000
25000
20000
15000
10000
5000
0
IgG1 (ng/mL)
*
OVA(1)
OVA(10)
OVA(100)
b
450
400
350
300
250
200
150
100
50
0
IgG2b (ng/mL)
*
*
OVA(1)
OVA(10)
OVA(100)

Figure S3

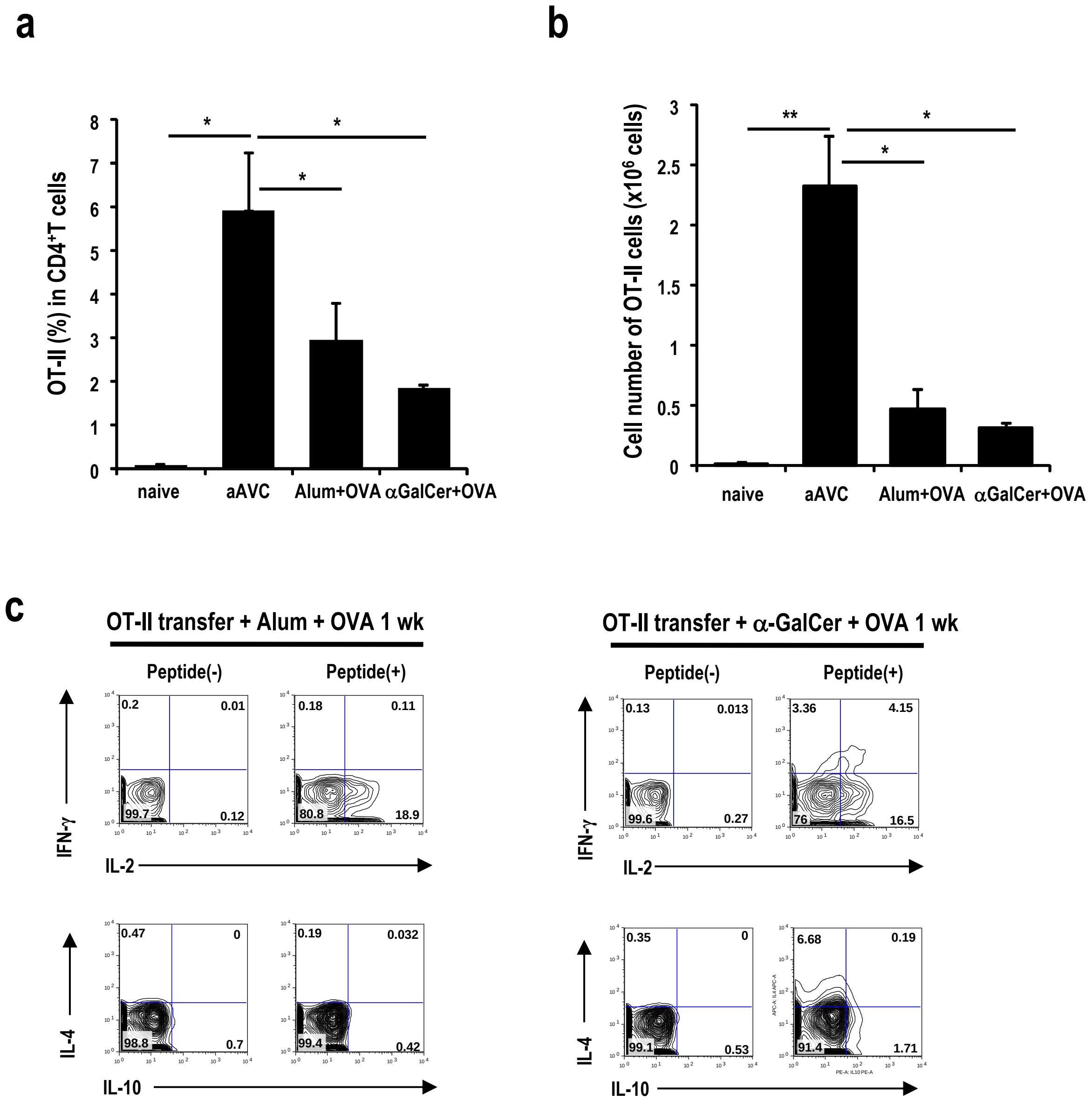

a
OT-II (%) in CD4+T cells
naive
aAVC
Alum+OVA
αGalCer+OVA
b
Cell number of OT-II cells (x10^6 cells)
naive
aAVC
Alum+OVA
αGalCer+OVA
c
OT-II transfer + Alum + OVA 1 wk
OT-II transfer + α-GalCer + OVA 1 wk
Peptide(-)
Peptide(+)
IFN-γ
IL-2
IL-4
IL-10

Figure S4

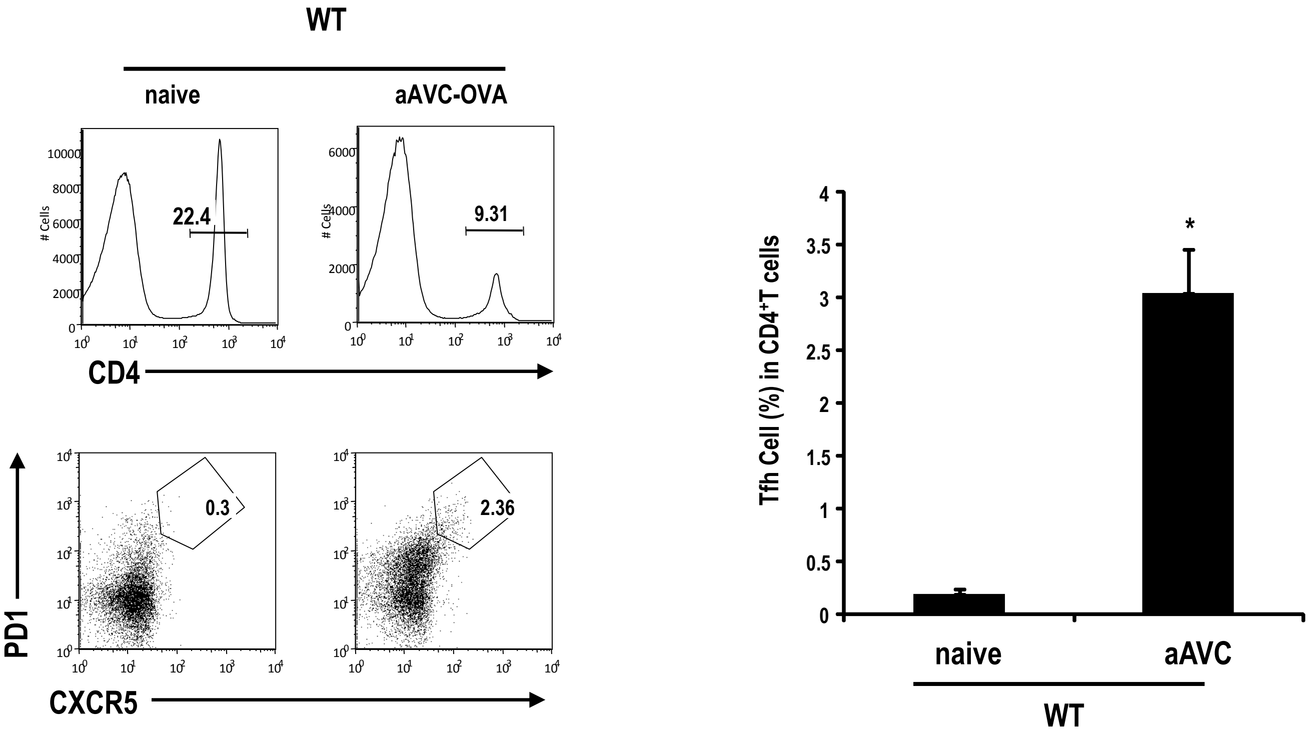

WT
naive
aAVC-OVA
Cells
22.4
9.31
CD4
PD1
CXCR5
0.3
2.36
Tfh Cell (%) in CD4+T cells
*
naive
aAVC
WT

Figure S5

a

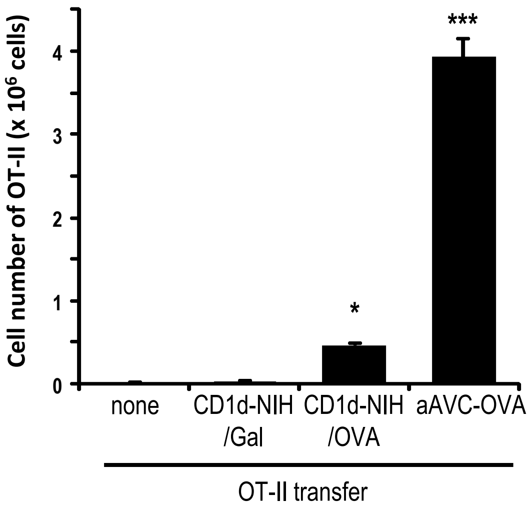

Cell number of OT-II (x 10^6 cells)
0
1
2
3
4
*
***
none
CD1d-NIH /Gal
CD1d-NIH /OVA
aAVC-OVA
OT-II transfer

b

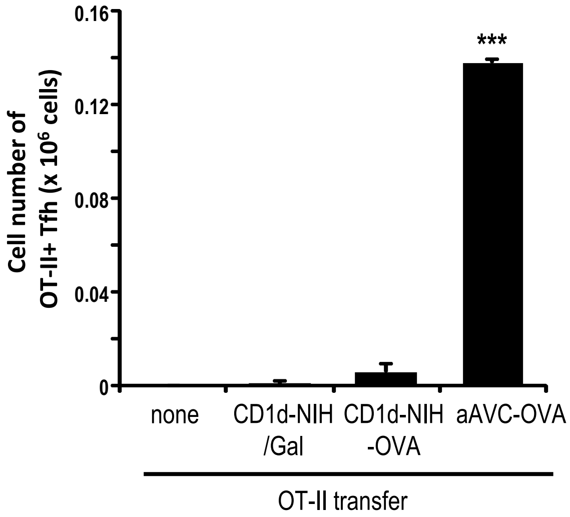

Cell number of OT-II+ Tfh (x 10^6 cells)
0
0.04
0.08
0.12
0.16
***
none
CD1d-NIH /Gal
CD1d-NIH -OVA
aAVC-OVA
OT-II transfer
